# One Health perspective on mycotoxins in poultry production: Ecology, toxicological effects, occupational and environmental exposure, food safety risks, and mitigation strategies (2020–2025)

**DOI:** 10.14202/vetworld.2026.2172-2207

**Published:** 2026-05-27

**Authors:** Nurgul Montayeva, Birzhan Nurgaliyev, Abzal Kereyev, Gaukhar Nagimova, Zhenis Kushmukhanov

**Affiliations:** West Kazakhstan Agrarian and Technical University named after Zhangir Khan, Uralsk 090009, Republic of Kazakhstan

**Keywords:** aflatoxins, climate change, food safety, masked mycotoxins, multi-mycotoxin contamination, occupational exposure, One Health, poultry production

## Abstract

Mycotoxins produced by toxigenic fungi remain a major challenge in poultry production and global food safety. Contamination of poultry feed with aflatoxins, ochratoxin A, fumonisins, deoxynivalenol, T-2 toxin, zearalenone, and other emerging mycotoxins is frequently reported worldwide, particularly under intensive production systems and changing climatic conditions. This review summarizes current evidence published between 2020 and 2025 on the occurrence, ecological drivers, toxicological effects, environmental and occupational exposure, food safety risks, analytical detection methods, and mitigation strategies of mycotoxins in poultry production within a One Health framework. Recent studies indicate that multi-mycotoxin contamination is common in poultry feeds, and emerging and masked mycotoxins may remain undetected by routine analytical approaches, thereby increasing the risk of underestimating exposure. Mycotoxins adversely affect poultry health through hepatotoxicity, nephrotoxicity, oxidative stress, immunosuppression, intestinal barrier disruption, microbiome dysbiosis, impaired reproductive performance, and reduced productivity. In addition, residues of several mycotoxins have been detected in meat and eggs, raising concerns regarding consumer safety. Airborne fungal spores and contaminated dust in poultry houses also represent important occupational hazards for poultry workers. Advances in analytical technologies, particularly Liquid Chromatography–Tandem Mass Spectrometry, biosensors, molecular diagnostics, and multiplex detection systems, have improved the sensitivity and reliability of mycotoxin monitoring. Various mitigation approaches, including feed hygiene management, adsorbents, probiotics, biological detoxification, and enzymatic degradation, have shown potential to reduce contamination and minimize toxic effects. However, the complete elimination of mycotoxins remains difficult due to the complexity of fungal ecology and the widespread occurrence of co-contamination. Overall, this review highlights the importance of integrated surveillance, improved feed management, advanced detection systems, and coordinated mitigation strategies within a One Health approach to reduce the impact of mycotoxins on poultry health, environmental safety, occupational exposure, and food security.

## INTRODUCTION

The poultry industry plays a critical role in ensuring global food security; however, its productivity is persistently compromised by contamination of feed and farm environments with toxigenic molds. Mycotoxins, which are low-molecular-weight secondary metabolites produced by fungi, represent a persistent biological hazard [1, 2]. Since the discovery of aflatoxin in the 1960s, following the outbreak of “Turkey X disease” in the United Kingdom, more than 400 mycotoxins have been identified, of which at least 30 have been confirmed to be toxic to poultry species [3, 4].

Feed ingredients such as maize, wheat, barley, and peanut meal are particularly susceptible to fungal colonization under tropical and subtropical conditions [5, 6]. In addition, climate variability, extreme weather events, shifting precipitation patterns, storage conditions, and globalized feed supply chain dynamics increasingly shape fungal ecology and drive the co-occurrence of multiple and masked mycotoxins in poultry feed ingredients [7]. The predominant genera, Aspergillus, Fusarium, and Penicillium, produce a wide range of metabolites, including aflatoxins (AFB_1_, AFB_2_), ochratoxin A (OTA), fumonisins (FB_1_–FB_3_), trichothecenes (deoxynivalenol, T-2 toxin), and zearalenone (ZEN). A more detailed classification and discussion of major and emerging mycotoxins in poultry feed are provided in the section “Common mycotoxins in poultry feed” to avoid redundancy and ensure structured synthesis. These compounds disrupt metabolic and immune functions, induce organ pathology, and impair growth and reproductive performance [8–10].

The economic losses associated with mycotoxins in poultry production are substantial and are estimated to exceed billions of US dollars annually due to feed rejection, reduced feed conversion efficiency, veterinary costs, and mortality. Beyond direct economic losses, mycotoxin contamination represents an emerging sustainability constraint. Recent life cycle assessment (LCA) analyses indicate that chronic mycotoxin exposure in broiler production may increase the carbon footprint by approximately 8%–9% due to reduced feed efficiency, higher resource consumption, increased greenhouse gas emissions, and elevated nutrient excretion. These findings highlight that feed contamination is not merely an economic and animal health issue but also a systems-level constraint that affects environmental sustainability and net-zero poultry production goals. Recent global monitoring programs, such as the DSM-Firmenich World Mycotoxin Survey 2025, continue to report a high prevalence of multi-mycotoxin contamination in poultry feed worldwide, confirming the sustained economic and environmental burden associated with compromised feed safety [9-17]. Beyond the decline in animal productivity, human exposure to mycotoxin residues in meat and eggs, as well as inhalation of contaminated dust in poultry facilities, raises serious concerns regarding public health and occupational diseases [18–20].

Recent evidence further indicates that emerging, masked, and modified mycotoxins represent an under-recognized challenge in poultry production systems. These toxin forms frequently coexist with regulated mycotoxins and may escape routine analytical detection while still retaining toxicological activity. Following ingestion, several masked mycotoxins can undergo hydrolysis in the gastrointestinal tract, releasing their parent toxic compounds, thereby increasing the likelihood of underestimation of exposure and health risks in poultry and humans [9, 14–16, 21]. Consequently, contamination profiles in commercial poultry feeds are increasingly characterized by complex multi-mycotoxin interactions rather than single toxin exposure scenarios. This dynamic of concealed exposure challenges current regulatory thresholds, conventional monitoring systems, and existing risk assessment frameworks, emphasizing the need for integrated surveillance and advanced non-targeted analytical strategies in poultry production systems.

Despite the growing body of literature on mycotoxins in poultry feeds, several important research gaps remain. Most previous studies have focused primarily on individual regulated mycotoxins, whereas limited attention has been paid to the ecological interactions, co-occurrence dynamics, and cumulative toxicological effects of multiple emerging and masked mycotoxins under field conditions. In addition, available reviews often emphasize poultry health, food safety, or analytical detection in isolation, with insufficient integration of occupational exposure, environmental dissemination, sustainability implications, and One Health perspectives. Furthermore, recent advances in biosensors, molecular diagnostics, high resolution mass spectrometry, and biological detoxification strategies have not been comprehensively synthesized alongside the rapidly evolving evidence regarding climate-driven fungal ecology and hidden contamination pathways. The lack of an integrated, up-to-date synthesis hampers the development of coordinated mitigation strategies to address the interconnected risks to animal health, environmental safety, occupational health, and food security.

Therefore, this review was conducted to synthesize current evidence published between 2020 and 2025 regarding the occurrence, ecological drivers, toxicological effects, exposure pathways, analytical detection methods, and mitigation strategies of mycotoxins in poultry production within a One Health framework. Particular emphasis was placed on multi-mycotoxin contamination, emerging and masked mycotoxins, occupational and environmental exposure pathways, and recent advances in monitoring and detoxification technologies to provide a comprehensive, up-to-date understanding of this evolving global challenge.

## REVIEW METHODOLOGY

### Literature search strategy

This review used a structured narrative approach to synthesize current evidence on mycotoxins in poultry production within a One Health framework. The review primarily focused on studies published between 2020 and 2025; however, selected high-impact reports and surveillance data available in early 2026 were also included to incorporate the most recent evidence on global contamination trends, toxicological risks, analytical advancements, and mitigation strategies associated with mycotoxins in poultry systems.

### Information sources and databases

A comprehensive literature search was performed using multiple international electronic databases, including PubMed, Scopus, Web of Science, ScienceDirect, and Google Scholar. Additional relevant publications were identified through manual searches of reference lists from eligible articles, review papers, international surveillance reports, and publications issued by recognized food safety and feed monitoring organizations.

### Search keywords and search combinations

The literature search was performed using combinations of relevant keywords and Boolean operators. The primary search terms included “mycotoxins,” “poultry,” “aflatoxins,” “ochratoxin A,” “fumonisins,” “deoxy-nivalenol,” “zearalenone,” “masked mycotoxins,” “emerging mycotoxins,” “multi-mycotoxin contamination,” “fungal ecology,” “feed safety,” “food safety,” “occupational exposure,” “One Health,” “biosensors,” “LC-MS/MS,” “biological detoxification,” and “climate change.” Different keyword combinations were adapted according to the indexing system and search structure of each database to maximize retrieval efficiency and relevance.

### Eligibility criteria

Studies were selected based on their scientific relevance to mycotoxin contamination and associated risks in poultry production systems. Emphasis was placed on studies evaluating the occurrence, ecology, toxicological effects, detection methods, environmental dissemination, occupational exposure, food safety implications, and mitigation strategies related to mycotoxins in poultry and poultry-derived products.

### Inclusion criteria

The inclusion criteria comprised: (i) original research articles, systematic reviews, meta-analyses, surveillance studies, and experimental studies published in peer-reviewed journals; (ii) studies investigating mycotoxin contamination in poultry feeds, poultry production environments, or poultry-derived food products; (iii) studies assessing toxicological, immunological, reproductive, microbiological, environmental, occupational, or food safety effects associated with mycotoxin exposure; (iv) studies examining analytical detection methods, surveillance systems, detoxification approaches, or mitigation strategies; and (v) studies relevant to the One Health perspective of mycotoxin contamination in poultry systems.

### Exclusion criteria

Conference abstracts lacking sufficient methodological details, duplicate publications, editorials, unpublished reports without accessible scientific validation, and studies unrelated to poultry production systems or mycotoxin-associated risks were excluded. Non-English articles without available translations and studies lacking adequate scientific relevance to the review objectives were also excluded.

### Study screening and selection

Retrieved publications were screened sequentially through title evaluation, abstract assessment, and full-text review. Studies meeting the predefined eligibility criteria were selected for inclusion. Duplicate records identified across databases were removed during the screening process. Priority was given to recent publications, large-scale surveillance studies, and studies providing comprehensive evidence regarding multi-mycotoxin contamination, emerging and masked mycotoxins, and One Health-related implications.

### Data extraction and synthesis

Relevant data extracted from the eligible studies included publication year, geographical location, study objectives, poultry species, mycotoxin type, contamination prevalence, ecological and environmental factors, toxicological findings, analytical detection methods, mitigation approaches, and implications for animal health, environmental safety, occupational exposure, and food safety. Particular attention was given to studies reporting co-occurrence of multiple mycotoxins, climate-associated fungal ecology, emerging and masked toxins, and recent advances in analytical and detoxification technologies.

### Thematic organization of the review

The collected evidence was narratively synthesized and organized into thematic sections covering fungal ecology and contamination pathways, occurrence and sources of mycotoxins, environmental dynamics, toxicological effects in poultry, food safety risks, occupational and environmental exposure, analytical detection methods, and mitigation and control strategies. This thematic structure was designed to provide an integrated understanding of the multifactorial nature of mycotoxin contamination in poultry production systems.

### One Health integration framework

The review was conducted within a One Health framework to emphasize the interconnected relationships among poultry health, environmental contamination, occupational exposure, and public health risks associated with mycotoxins. The synthesis specifically considered the role of fungal ecology, feed contamination, environmental dissemination, and human exposure pathways in shaping the broader impact of mycotoxins on sustainable poultry production and global food safety.

## OCCURRENCE AND SOURCES OF MYCOTOXINS IN POULTRY PRODUCTION SYSTEMS

### Fungal ecology and routes of contamination

The geographical distribution of molds and mycotoxins in poultry production varies by region and is closely linked to feed sources. Climate-driven shifts in agroecological zones are increasingly associated with the emergence of new geographic risk areas, including the northward expansion of Fusarium species and heightened aflatoxin susceptibility in regions experiencing prolonged heat and drought stress, thereby altering traditional contamination patterns in poultry feed supply chains [17, 22]. Aflatoxins produced by Aspergillus spp. show well-documented prevalence in Southeast Asia and Sub-Saharan Africa, highlighting region-specific mycotoxin risks in poultry feed [23]. In Russia, toxigenic cereal-associated fungi, including various Fusarium and Alternaria species, have been reported, underscoring mold contamination in feed materials [24]. Similarly, Canada has reported diverse Alternaria isolates on cereal crops, indicating widespread geographical mold contamination of feed grains used in poultry production [25].

Mycotoxin contamination of poultry feed exhibits distinct geographical patterns driven by grain supply chains, storage practices, and climatic conditions, and regional studies frequently report the co-occurrence of multiple mycotoxins. A synthesized overview of global prevalence trends and regional distribution patterns is summarized in Table 1 [9, 17, 26–35], integrating recent multi-regional monitoring data on major mycotoxins in poultry feeds. For example, up to nine mycotoxins, including aflatoxins, were detected in feed samples from Navarra, Spain [31]. The influence of global and regional climate further shapes the prevalence and concentration of mycotoxins in feed across all continents, including the Middle East and North Africa region [36]. Emerging evidence further links precipitation variability, temperature extremes, and crop stress to altered mycotoxin co-occurrence patterns and the emergence of new toxin combinations in feed materials, thereby complicating risk prediction and management in poultry production systems [7]. In addition, climate-associated changes in maize used for poultry feed have been documented in Serbia, emphasizing the role of environmental factors in modulating mycotoxin risk [37].

**Table 1:** Regional mycotoxin patterns in poultry feeds across multiple surveys.

Region/country	Key pattern in poultry feeds	Reference
Global summary	AFs, ZEN, DON, fumonisins, OTA, T-2, HT-2, plus emerging toxins. Multiple regulated and emerging toxins per sample are common	[9, 27, 30, 34]
Sub-Saharan Africa	High AF; frequent co-occurrence of AF + fumonisins + trichothecenes; many samples >20 µg/kg AF	[17, 28, 32, 33]
Pakistan (Punjab)	All samples contained AFB1 + FB1; 73% exceeded European Commission limits for AFB1; 3–14 mycotoxins/sample	[35]
Kenya (broiler farms)	100% contaminated; 93% contained >3 mycotoxins; fumonisins (93%) and DON (88%) dominant	[17]
Romania feed mill	Broiler feed contained DON, ZEN, and fumonisins in >75%–95% of samples, usually below European Union limits but with frequent co-occurrence	[29]
Spain (Navarra)	Poultry feed with DON and ZEN commonly detected; most samples below European Union limits, but 63.5% contained 2–5 mycotoxins	[31]
Saudi Arabia	100% of compound poultry feeds contaminated with at least two mycotoxins; AFs detected in 84% of samples	[26]

Collectively, these findings indicate that mold- and mycotoxin-related risks are global yet geographically heterogeneous, necessitating region-specific feed monitoring and post-harvest management strategies to reduce exposure in poultry production systems. Recent global feed monitoring surveys and multi-regional assessments consistently indicate a high prevalence of mycotoxin contamination in poultry feeds worldwide, with aflatoxins predominating in tropical and subtropical regions, Fusarium-derived toxins (DON, ZEN, fumonisins) more frequently reported in temperate cereal-based feed systems, and OTA commonly associated with storage-related contamination across diverse climatic zones. These global patterns highlight not only the widespread occurrence of mycotoxins but also their frequent co-occurrence in feed ingredients and finished feeds, reinforcing the need for region-specific surveillance and integrated risk assessment in poultry production systems [14, 15, 38].

Mold fungi colonizing poultry feed primarily originate from cereal grains and by-products used in feed formulations, as previously discussed in the context of fungal ecology and regional contamination patterns. The dominant toxigenic genera involved in feed contamination are Aspergillus, Fusarium, and Penicillium, which have well-established associations with major mycotoxins such as aflatoxins, ZEN, OTA, fumonisins, and trichothecenes, including DON and T-2 toxin [26, 39–41]. To avoid redundancy, the ecological roles of these genera are summarized here with emphasis on their contribution to multi-mycotoxin contamination rather than repeated taxonomic description.

In particular, Aspergillus species, especially A. flavus and related members of section Flavi, are the principal producers of aflatoxins and are frequently detected in cereal-based poultry feeds. Molecular characterization and surveillance studies confirm a persistent risk of contamination in regions with warm and humid climates [26, 40, 41]. Fusarium species are key producers of trichothecenes and ZEN, and their mycotoxins, including DON, T-2, HT-2, ZEN, and fumonisins, are commonly detected in feeds. Ecological and climatic factors strongly influence their prevalence and co-occurrence with other mycotoxins [34, 39, 42].

Penicillium species also contribute to feed contamination, particularly under storage conditions favorable to mold growth, and in certain contexts are implicated in the production of OTA [26, 41]. Overall, the ecology of these molds is closely linked to grain origin, moisture content, surface area exposure during processing, and storage conditions, including temperature, humidity, and storage duration [39, 40, 43].

Models of co-occurrence of multiple mycotoxins in feed are increasingly documented, reflecting complex fungal communities and synergistic or antagonistic interactions among fungal metabolites. Recent high-throughput analytical surveys using multi-mycotoxin LC-MS/MS approaches have reported the simultaneous detection of numerous mycotoxins within single feed samples, reflecting complex fungal ecosystems and the ecological reality of multi-toxin exposure in commercial poultry production systems [9, 17].

Co-occurrence analyses report the presence of nine or more mycotoxins in some feeds and indicate that aflatoxins frequently appear alongside OTA, ZEN, DON, and fumonisins, posing potential additive or synergistic risks to poultry health and food safety [40, 44, 45]. The ecological reality of co-contamination is further supported by regional studies showing high exposure to multiple mycotoxins in poultry feeds across Europe, Africa, and Asia [3, 40, 44]. These patterns emphasize the need to consider mold ecology and mycotoxin risk as a multi-toxin, ecosystem-level problem rather than an issue of individual toxins [44, 45].

### Pre-harvest contamination

Pre-harvest contamination arises from mold colonization of cereal crops in the field, influenced by climatic conditions such as heat and humidity that favor Fusarium, Aspergillus, and related genera. Mycotoxin risk depends on crop species, cultivar resistance, harvest timing, and pre-harvest storage practices, with DON, ZEN, and aflatoxins often associated with field-accumulated mold in cereal feed [3, 40, 41]. Extreme climatic events, including drought stress, flooding, and elevated atmospheric CO_2_, have recently been linked to shifts in toxin biosynthesis pathways and increased multi-mycotoxin co-occurrence in cereals used for poultry feed, highlighting the need for climate-resilient agricultural and feed safety strategies.

Seasonal and regional studies report variability in mycotoxin prevalence in maize and other cereals used for poultry feed, consistent with climate-driven differences in fungal ecology during the pre-harvest stage [3, 7, 40, 46]. Emerging datasets also highlight seasonal spikes in mycotoxin occurrence, including increased Fusarium-associated toxins during cooler storage periods and winter microclimatic instability in enclosed poultry systems, emphasizing the need for seasonally adaptive monitoring strategies.

### Post-harvest contamination and storage-associated risks

Post-harvest factors, such as grain moisture, temperature, aeration, storage duration, and packaging, determine mold growth and mycotoxin production in stored feeds and feed ingredients. Studies have consistently documented a high prevalence of mycotoxins in complete poultry feeds over multiple years, with DON detected in a subset of samples, illustrating how storage and processing conditions sustain mold activity and toxin formation even after harvest [40]. In other regions, storage conditions combined with cereal-based feed ingredients, particularly maize/corn, favor the accumulation of OTA and ZEN, highlighting the close relationship between ingredient origin, storage microclimate, and fungal ecology in feeds [40, 41].

Hygienic practices and farm conditions further influence post-harvest contamination. Poor storage, moisture ingress, and cross-contamination at feed mills and storage facilities promote fungal proliferation and transfer of toxins into finished feeds [19, 47].

The risk of contamination extends throughout the supply chain, from farm intake to finished feeds. Industrial and semi-industrial poultry production systems create opportunities for the spread of fungal spores and metabolites via bioaerosols, particularly in high-density housing systems where feed slurry, dust, and aerosols contain mold propagules and mycotoxigenic fungi. The One Health perspective emphasizes the interconnections among feed contamination, animal, and human health across the food chain [19, 43]. Regional syntheses further highlight how locally produced feeds and imported ingredients introduce diverse fungal communities and mycotoxins into the poultry feed chain, underscoring the importance of supply chain monitoring and ingredient quality verification [44, 48, 49].

During grinding, milling, pelleting, and mixing, contamination can spread through soiled equipment, dust, and inadequate cleaning, allowing molds already present in the ingredients to proliferate and produce secondary metabolites in the finished feed [43, 47]. On-farm processing, storage, and feeding conditions can reintroduce moisture, promote mold growth on exposed grains, and facilitate bioaerosol exposure to both poultry and workers. Systematic reviews highlight that the farm environment itself contributes to microbial and fungal contamination of feed matrixes [19, 47].

When feeds are stored in silos or bins with high moisture content, mold spores can colonize exposed feed surfaces, particularly when temperature and RH are not strictly controlled [40, 43]. Interventions such as targeted adsorbents and process modifications are critical at multiple stages of the production chain to mitigate contamination [39, 50, 51].

## COMMON MYCOTOXINS IN POULTRY FEED

Cereal- and grain-based ingredients used in poultry feeds are frequently contaminated with mycotoxins produced by filamentous fungi. The most commonly reported and concerning mycotoxins in poultry feeds include aflatoxins, primarily aflatoxin B1 (AFB1), OTA, ZEN, and trichothecenes such as DON, along with its derivatives T-2 and HT-2, as well as fumonisins (FB1, FB2) [31, 38, 52]. In addition, regional studies and reviews document the co-occurrence of multiple mycotoxins in feeds and feed ingredients, highlighting the complexity of exposure experienced by poultry under real-world feeding programs [31, 41, 44]. This synthesis draws on extensive reviews, regional monitoring, and toxin-focused research to characterize the spectrum of mycotoxins commonly found in poultry feeds, their potential health and production impacts, and strategies for monitoring and mitigation.

### Aflatoxins

Aflatoxins (AF), particularly AFB1, are among the most extensively studied mycotoxins in poultry feed due to their hepatotoxicity, carcinogenicity, and potential transfer into eggs and meat. Their presence in feed remains a persistent concern across all regions and feed types [38, 41, 44, 52]. Regulatory and monitoring frameworks highlight aflatoxins as high-priority contaminants in poultry supply chains [53].

### Ochratoxin A

OTA is a common contaminant in cereals and cereal-based poultry feeds and is associated with nephrotoxicity and oxidative stress in birds. OTA can accumulate in tissues and be transferred to animal products, raising concerns for consumer safety [38, 44, 53, 54]. Recent European risk assessments and global reviews confirm that OTA remains a persistent feed hazard and a priority for mitigation [44, 55].

### Zearalenone

ZEN is an estrogenic mycotoxin that can disrupt reproductive physiology in poultry. ZEN frequently co-occurs with other mycotoxins in cereals and feed ingredients and is regularly detected in poultry feed studies [31, 38, 41, 52].

### Trichothecenes

DON and other trichothecenes, including T-2 and HT-2, can impair productivity and reduce feed intake and weight gain in poultry at certain exposure levels. DON is often found in combination with other mycotoxins in feeds and feed ingredients [31, 41, 52].

### Fumonisins

Fumonisins (FB1 and FB2) are another significant group of mycotoxins of concern in poultry feeds. Studies link fumonisins to adverse production outcomes and potential impacts on egg safety. Co-occurrence with DON and ZEN has been documented in feed surveys [31].

### Emerging and modified mycotoxins

Beyond traditionally regulated mycotoxins, increasing attention is being directed toward emerging and modified forms that frequently co-occur with major toxins in poultry feeds. Emerging Fusarium metabolites such as enniatins, beauvericin, moniliformin, and Alternaria toxins, including alternariol and alternariol monomethyl ether, are increasingly reported in global feed monitoring surveys. Recent large-scale surveillance programs (2024–2025) indicate that these metabolites often coexist with regulated toxins such as DON, ZEN, fumonisins, and aflatoxins, contributing to complex multi-mycotoxin exposure scenarios in poultry production systems [14, 15, 28].

Enniatins and beauvericin are cyclic hexadepsipeptides with ionophoric properties that can disrupt cellular membranes, induce oxidative stress, and interfere with mitochondrial function. Although poultry-specific in vivo toxicity data remain limited, experimental evidence suggests potential immunomodulatory, cytotoxic, and reproductive effects, particularly under co-exposure conditions. Moniliformin has been associated with cardiotoxic and growth-depressing effects in other animal models; however, data in poultry remain insufficient for reliable risk characterization. Alternaria toxins, including alternariol, exhibit genotoxic and endocrine-disrupting potential, raising additional concerns regarding chronic low-dose exposure in laying hens and broilers [38, 56].

Masked and modified mycotoxins represent an additional layer of complexity. Conjugated forms such as deoxynivalenol-3-glucoside, zearalenone-14-glucoside, and hydrolyzed or matrix-bound fumonisins may evade detection by conventional targeted analytical methods. Importantly, these modified forms can undergo hydrolysis in the gastrointestinal tract, releasing the parent toxin and thereby amplifying systemic exposure. Consequently, reliance on standard assays may underestimate the true toxicological burden in poultry and potential residue transfer into edible tissues and eggs [9, 57].

Feeds may also contain a broader spectrum of mycotoxins, including sterigmatocystin, and recent reviews emphasize the diversity of toxins that can contaminate cereal-based poultry feeds [41, 44].

## MULTI-MYCOTOXIN CO-OCCURRENCE PATTERNS

Building on these observations, co-occurrence of multiple mycotoxins in poultry feeds is now recognized as the ecological norm rather than the exception. Recent large-scale feed surveys increasingly report the simultaneous presence of emerging and masked mycotoxins alongside regulated toxins, highlighting that real-world poultry feeds rarely contain a single toxin but rather complex, partially hidden contamination profiles [9]. Regional studies frequently report the simultaneous presence of several mycotoxins, including AF, OTA, ZEN, DON, FB, and others, and document correlations between contaminants in feed ingredients and finished feeds [31, 41]. Recent global monitoring surveys (2024–2025), including large-scale industry datasets such as the DSM-Firmenich World Mycotoxin Survey and Selko global reports, confirm persistent multi-mycotoxin co-occurrence and increasing detection of masked and emerging forms, underscoring the dynamic and evolving nature of contamination patterns in poultry feed supply chains [14, 15]. Such combined exposure may exert additive or synergistic negative effects on poultry health and productivity, complicating the development of mitigation strategies [44].

Regional and seasonal variations further modulate exposure risk. For example, feed studies in Nigeria demonstrate seasonal fluctuations in mycotoxin levels in feeds and ingredients, highlighting the influence of climate on contamination profiles and the need for seasonal monitoring and management [53]. Similar patterns have been observed in other regional reports [54].

**Figure 1:**
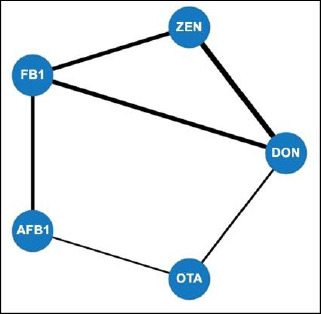
Representative co-occurrence network of major mycotoxins detected in poultry production systems based on recent poultry feed surveys and review data (2020–2025). Node size indicates the relative importance and frequency of detection of individual mycotoxins, whereas edge thickness reflects the reported likelihood of co-occurrence in feed and production environments. The network primarily represents commonly reported regulated toxins (AF, DON, ZEN, FB, OTA), although recent surveys suggest that emerging and masked mycotoxins may also co-occur within complex multi-mycotoxin contamination profiles.

In line with the aforementioned evidence, a representative co-occurrence network of major mycotoxins typically detected in poultry production systems was constructed (Figure 1). The network synthesis was derived from published poultry feed surveys, regional monitoring studies, and recent systematic reviews reporting multi-mycotoxin detection patterns in feed ingredients and complete feeds. The network illustrates commonly reported combinations of mycotoxins in poultry feed and production environments. Node size reflects the relative frequency of detection, whereas edge thickness represents the likelihood of simultaneous occurrence, as documented in recent poultry surveys and reviews. Fusarium-derived toxins (DON, ZEN, FB_1_) frequently co-occur, whereas aflatoxins and OTA often appear in combination with fumonisins or DON, highlighting the multifactorial exposure risk in poultry production.

Table 1 summarizes representative regional investigations illustrating the geographic heterogeneity and consistent multi-mycotoxin co-occurrence patterns observed in poultry feeds worldwide. The data demonstrate marked geographical variability in contamination profiles while consistently highlighting the widespread presence of multi-mycotoxin mixtures.

Worldwide monitoring of approximately 13,800 livestock feed samples, including 5,363 poultry samples, showed that mycotoxins are nearly ubiquitous, usually at low-to-moderate levels, with occasional exceedances of regulatory limits. Asia contributed the highest proportion of feed data, followed by Africa, Europe, and Brazil [30].

Sub-Saharan Africa reports frequent high-level contamination, especially with AFs and fumonisins, linked to warm, humid climates and weak feed control systems [17, 28, 32, 33]. Europe generally shows lower contamination levels, with only 0%–2.5% of compound feeds exceeding European Union limits despite common detection of DON and ZEN [31]. Middle Eastern data, including Saudi Arabia and Iraq, show 100% contamination of poultry feeds, although usually within national limits, with AFs, FB1, DON, ZEN, and OTA commonly co-occurring [22, 58].

In Sub-Saharan Africa, AF frequently occurs at elevated concentrations, often exceeding 20 µg/kg, and is commonly co-contaminated with fumonisins and trichothecenes. In Pakistan (Punjab), all analyzed samples contained both AFB1 and FB1, with a high proportion exceeding European Commission limits for AFB1 and multiple toxins (3–14 per sample) detected simultaneously. Kenyan broiler feed surveys report universal contamination, with fumonisins and DON predominating and most samples containing more than three mycotoxins [17, 28, 32, 33, 35].

European surveys show high detection frequencies of DON, ZEN, and fumonisins in broiler feeds from Romania and Spain (Navarra). Although concentrations are generally below European Union regulatory limits, co-occurrence is common, with a substantial proportion of samples containing two to five mycotoxins. In Saudi Arabia, compound poultry feeds were universally contaminated with at least two mycotoxins, and aflatoxins were detected in most samples [22, 29, 31].

Overall, these regional patterns underscore the global nature of poultry feed contamination and the predominance of multi-mycotoxin exposure scenarios, even when individual toxin levels remain within regulatory thresholds.

## ENVIRONMENTAL DYNAMICS AND FUNGAL ECOLOGY IN POULTRY SYSTEMS

### Climatic and environmental factors

Poultry production occurs within artificially controlled environments where microclimate, feed substrates, litter, bedding materials, and airborne particles create conditions conducive to mold growth and mycotoxin formation. The importance of this issue is highlighted by the One Health concept, which recognizes that environmental conditions on poultry farms influence fungal ecology, animal health, and potential human exposure through contaminated products and farm dust [19].

Recent evidence further emphasizes that environmental factors, such as litter materials and indoor microclimate, modulate mold exposure, particularly Aspergillus spp., and associated mycotoxins, thereby affecting broiler health and productivity [59, 60]. Seasonal variability further modulates fungal ecology in poultry systems, with several recent studies reporting seasonal spikes in fungal load and mycotoxin levels in litter, dust, and feed matrixes, particularly during colder periods when reduced ventilation, higher indoor humidity, and prolonged feed storage may promote mold persistence in poultry houses [7, 46].

The interaction between climate change and intensive poultry production systems may amplify mycotoxin risks by altering temperature and humidity patterns in poultry houses, potentially increasing fungal activity and toxin formation in feeds and the environment [61, 62]. Recent forward-looking studies (2024–2025) indicate that climate change is expected to substantially reshape mycotoxin risk profiles in poultry feed systems through increased frequency of extreme weather events, poleward migration of toxigenic fungi, and elevated atmospheric CO_2_ levels that influence fungal metabolism and toxin biosynthesis. Predictive climate–mycotoxin models suggest potential increases of 10%–30% in selected toxin concentrations by 2030 under warming and drought-prone scenarios, particularly for aflatoxins in warmer regions and for Fusarium-derived toxins in temperate zones. These climate-driven shifts are likely to alter fungal community composition and increase the frequency of multi-mycotoxin contamination across feed and environmental matrixes [5, 14, 15, 37].

Environmental monitoring using IoT technologies is being explored as a practical approach to track key climate and air quality variables in large-scale enclosed poultry facilities, aiming to reduce mold- and toxin-related risks [63]. Integration of real-time IoT monitoring with predictive climate analytics could enable dynamic mycotoxin risk management by enabling early detection of microclimatic conditions favorable for fungal growth and facilitating adaptive interventions in feed storage, ventilation, and sourcing strategies [64]. Collectively, climate-driven processes and local environmental conditions shape fungal ecology and mycotoxin risk in poultry production systems [60-63].

### Temperature and humidity-associated fungal dynamics

Temperature and its extremes are major factors promoting mold colonization and mycotoxin synthesis in feed substrates. Empirical data show that mold abundance correlates with elevated temperatures, with thresholds such as Tavg ≥30°C, Tmax≥32°C, and Tmax≥35°C, whereas toxin production is associated with extremely high daily maximum temperatures (Tmax ≥35°C) under certain conditions [62]. This pattern aligns with broader observations that heat and dryness can favor the growth of specific mycotoxin-producing fungi in agricultural substrates and feed ingredients used in poultry production [64, 65]. Specifically, climate-related temperature regimes have been shown to influence the prevalence and activity of mycotoxins in feeds and feed materials in regional contexts [64, 65].

RH and atmospheric drought interact with temperature, thereby influencing mold ecology. In the same study that identified temperature thresholds, a low minimum RH (≤40%) was highlighted as a predictor of mold abundance, indicating that dry, hot conditions during storage or transport can promote mold growth in feed matrixes used in poultry production [62]. This interplay between thermal stress and humidity is reflected in mycotoxin occurrence reviews under geoclimatic variability, where temperature and humidity dynamics repeatedly emerge as key determinants of mold proliferation and mycotoxin production in agricultural systems [64, 65]. Recent reviews and monitoring studies (2024–2025) further corroborate the climate–mycotoxin relationship, emphasizing the role of geoclimatic variability and environmental stressors in shaping fungal ecology and toxin diversity in feed and poultry production environments [14].

### Geoclimatic influences on mycotoxin risk

Geoclimatic and regional climatic conditions shape mycotoxin risk in feeds. Geographic and climatic factors establish the baseline risk of mycotoxin synthesis by fungi such as Fusarium and Aspergillus spp. in poultry feeds, with temperature often the dominant factor. Regional analyses indicate that ambient temperature is strongly associated with fungal metabolite production, including mycotoxins, under field conditions where poultry feed crops are cultivated [64]. These findings emphasize the importance of considering climatic context when assessing mycotoxin risk in poultry feeds [66].

Climate change projections consistently support the need for climate-informed predictive modeling and adaptive feed management strategies within a One Health framework, particularly given the role of seasonal fluctuations in temperature, humidity, and storage microclimates in driving multi-mycotoxin co-occurrence [15].

AFB1 remains a major concern for poultry nutrition due to its severe health and productivity impacts. The climate–aflatoxin risk relationship is supported by analyses of AFB1 dynamics along the feed-to-food chain, which demonstrate that environmental conditions during crop growth and storage influence AFB1 production and subsequent exposure through animal-derived products [66]. Systematic reviews of trichothecenes and other mycotoxins further identify climate as a driver of mycotoxin diversity and prevalence in poultry feeds [34].

### Litter materials and microclimate

Litter is a key environmental factor determining moisture, ammonia production, and microclimate in poultry houses. Wood shavings, in particular, have been identified as potential sources of fungal contamination, which can affect respiratory health and lead to mold colonization under favorable climatic conditions. Temperature elevation and humidity fluctuations can interact with litter, thereby modifying fungal growth dynamics and infection risk [61]. More broadly, environmental factors in poultry houses indicate that microclimatic characteristics play a critical role in mold development and can be managed through litter selection, ventilation, and husbandry practices [67].

### Airborne particles, fungi, and microclimate dynamics in poultry houses

Poultry operations generate airborne PM that can carry fungal spores, including Aspergillus spp., thereby posing occupational hazards and animal health risks. Recent studies identify intensive poultry farms as potential reservoirs of azole-resistant Aspergillus species, raising important One Health concerns related to occupational exposure, antifungal resistance, and fungal bioaerosol loads within enclosed housing systems [14, 59]. Systematic attention to PM and Aspergillus spp. has been highlighted as a priority for surveillance in poultry systems todue of their roles as environmental reservoirs and exposure pathways for both animals and humans [59]. This connection between environmental particles, fungal ecology, and health outcomes underscores the need for careful microclimate and air quality management to reduce mold-related risks on poultry farms [61].

Empirical observations of aspergillosis and aerosacculitis outbreaks in poultry demonstrate that a combination of environmental factors, including excess ammonia, high litter moisture, temperature fluctuations, and decomposing bedding, creates favorable conditions for Aspergillus colonization in broilers [60]. These factors interact with the house microclimate and can be exacerbated by climate-driven changes in indoor and outdoor temperature, highlighting the importance of integrated environmental management in poultry housing [60].

### Microclimate dynamics and monitoring systems

The microclimate in uninsulated poultry houses and other poultry facilities is shaped by temperature, humidity, ventilation, and heat load. Understanding and characterizing this dynamic is crucial for predicting mold risks and implementing effective mitigation strategies [67]. Modern sensor technologies and automation, including Internet-of-Things-based environmental monitoring, provide practical tools for real-time data collection on climate and air quality, thereby enabling proactive management of mold risk in poultry operations [63].

### Fungal communities in litter and air

Fungal communities in poultry litter and air exhibit considerable diversity. Litter commonly harbors genera such as Aspergillus, Penicillium, Cladosporium, and Alternaria. Air samples from broiler houses contain airborne fungi, including Trichosporon, Candida, Aspergillus, Cladosporium, and Alternaria, with evidence indicating that fungal aerosol diversity increases as broilers age [68].

### Management activities and aerosolization

Daytime management activities, particularly during feed distribution, are associated with spikes in fungal dust, releasing fungi such as Penicillium, Mucor, and Aspergillus into the air [69]. The litter environment serves as a reservoir for microscopic fungi, with pronounced seasonal fluctuations affecting fungal communities in litter, dust, and air, including potential winter-associated increases in fungal persistence due to reduced ventilation and microclimatic humidity in enclosed poultry houses [43, 70].

Aspergillus species, especially A. fumigatus, are linked to aerosacculitis and aspergillosis in poultry. Specific isolates have been recovered from the lungs of affected birds as well as from the surrounding environment [60, 71]. Poultry houses are significant sources of bioaerosols, with fungal spore emissions varying depending on housing type [72-74].

Modern air sampling technologies, such as Coriolis µ samplers, enhance the detection of fungal contamination in poultry facilities [75]. Effective litter management strategies can modulate the respiratory fungal microbiome and reduce mold-related disease risks on farms [76]. Phenotypic characterization of pathogenic fungi in Egyptian poultry farms has highlighted their potential impact on animal health [77].

### Interaction with microbial and feed factors

The chemical composition of poultry feeds strongly influences mold growth and mycotoxin risk, determining substrate quality and the presence of mycotoxin-producing fungi in feed ingredients. Importantly, environmental factors such as litter microclimate, airborne fungal exposure, and feed contamination interact with the poultry gut microbiome, creating a dynamic environmental–microbial axis that modulates mycotoxin metabolism, intestinal health, and overall host susceptibility to toxin-related stress [19, 76]. Feed mycobiomes vary depending on ingredient composition, processing, and storage conditions [78-80].

The intestinal microbiota can biotransform mycotoxins, generating less harmful metabolites or, in some cases, more toxic forms, thereby modulating both chick health and toxin exposure [81]. Environmental exposure to fungal spores and mycotoxin-contaminated feed may also reshape gut microbial composition, influencing detoxification pathways, intestinal barrier integrity, and immune responses in poultry under intensive production conditions [81].

Antibiotic-free feeding strategies using phytogenics and plant polyphenols, such as catechins, hops extracts, and peppers, probiotics (Levucell SB), prebiotics/inulin with Lactiplantibacillus spp., and yeast fractions can modulate gut ecology, strengthen the intestinal barrier, and reduce colonization by mycotoxigenic fungi [82-88].

Feed adsorbents and antioxidants directly reduce mycotoxin exposure by binding or neutralizing toxins. Activated charcoal and antioxidant/adsorbent-based strategies have been shown to lower the risk of aflatoxicosis in broilers [89, 90].

Given concerns over AMR associated with antibiotic use, integrating feed composition optimization with microbiome-targeted additives is crucial for sustainable poultry production and effective mycotoxin control [91, 92].

## TOXICOLOGICAL EFFECTS OF MYCOTOXINS IN POULTRY

### General pathophysiology

The general pathophysiology of mycotoxins in poultry involves hepatotoxicity, nephrotoxicity, immuno-suppression, and intestinal barrier disruption, largely mediated by oxidative stress, inflammatory signaling, ER stress, and dysregulated xenobiotic metabolism. Oxidative stress represents a convergent mechanistic pathway across diverse mycotoxin classes, linking hepatic injury, immune dysregulation, and barrier dysfunction [15, 93–97].

Aflatoxins cause liver damage and immune suppression. Chronic exposure in laying hens leads to biochemical and histopathological changes in the liver and kidneys, which can be mitigated by ethoxyquin or N-acetylcysteine and further alleviated through probiotics and targeted interventions along the microbiota–gut–liver axis [93, 98, 99].

OTA induces oxidative stress and redox imbalance in liver and kidney tissues. Dietary selenomethionine modulates OTA-induced transcriptomic changes, whereas interactions with CYP450 influence OTA toxicity [94, 100]. OTA-associated gut dysbiosis further amplifies disruption of the gut–liver axis, thereby contributing to systemic damage [96].

FB1 triggers ER stress and inflammatory signaling and disrupts CYP450 function, thereby promoting liver injury in poultry models, including young quails [97].

T-2 toxin causes toxic damage to the liver and kidneys. Sodium butyrate mitigates its effects via Nrf2 signaling and CYP450 modulation in quails [101].

Citrinin primarily induces nephrotoxicity, as confirmed by histopathological analysis of poultry kidneys [95].

### Effects on growth and performance

Mold contamination of poultry feed with mycotoxins such as aflatoxins, DON, fumonisins, OTA, and ZEN poses significant risks to broiler growth, immunity, and food safety [10, 102, 103]. Adverse effects are observed even at subclinical doses or with toxin mixtures, with repeated exposure leading to reduced body weight gain and impaired feed conversion [104–106]. Modern poultry production systems are predominantly characterized by chronic exposure to low-to-moderate concentrations of multiple co-occurring mycotoxins, which, even below regulatory limits, may cumulatively impair nutrient digestibility, gut integrity, immune competence, and vaccine responsiveness in broilers and layers [10, 68].

Gut health is central to these effects. Combined exposure to fumonisins and DON disrupts cecal microbiota and intestinal morphology, thereby increasing susceptibility to necrotic enteritis [107]. Co-contamination frequently results in additive or synergistic effects that exceed the impact of individual toxins alone [104]. This risk is further amplified by masked and modified mycotoxins that may be reconverted to their parent toxic forms in the gastrointestinal tract, thereby contributing to underestimated cumulative toxicity [9].

Molds such as Aspergillus, Penicillium, and Fusarium produce major mycotoxins, including aflatoxins, OTA, trichothecenes, fumonisins, and ZEN, which can reduce egg production, alter egg composition and shell strength, and leave residues in eggs. The severity of effects depends on dose, age, and toxin type and can be partially mitigated using binders, detoxifiers, and management strategies.

### Egg mass and laying rate

Feed containing AFB1, DON, and OTA reduced overall egg mass and laying rate while increasing the feed-to-egg ratio in a 12-week study; the use of binders mitigated these reductions. Eggshell strength declined by approximately 12% after 12 weeks of combined AFB1/DON/OTA exposure, and binders prevented this decrease [108].

### Dose-dependent effects

High levels of AFB1 (546 µg/kg feed) reduced laying performance in hens, whereas moderate levels caused less pronounced or variable effects [109].

### Immunotoxic and hematological effects

Mold contamination and associated mycotoxins, particularly AFB1 and trichothecenes such as DON, suppress poultry immunity and disrupt hematological parameters. Exposure to AFB1 causes atrophy of lymphoid organs and lymphocyte depletion, thereby undermining cell-mediated immunity and vaccine responses [110, 111]. Mycotoxins induce oxidative stress and impair liver and kidney function, thereby altering hepatic enzyme activity and blood coagulation profiles [112, 113].

DON promotes Campylobacter jejuni proliferation by compromising intestinal integrity. Co-exposure to multiple mycotoxins enhances immunosuppression, even at low levels, as confirmed by studies on multi-toxin contaminated poultry feed [28, 114].

Both acute and chronic OTA exposure reduce total circulating leukocytes and cause heteropenia and lymphopenia, indicating systemic immunosuppression and increased disease susceptibility [10].

Chronic exposure to trichothecenes and Fusarium toxins decreases vaccine titers and impairs antibody responses, although the effects vary by toxin type and dose [115].

DON and other trichothecenes disrupt cytokine signaling both in the gut and systemically, increasing pro-inflammatory mediators, altering mucosal defense, and slowing recovery after intestinal infections [115, 116].

In the bursa of Fabricius and other lymphoid tissues, mycotoxins induce cell depletion and histopathological lesions. Atrophy correlates with decreased cellular immunity [10].

Some studies report altered platelet counts and changes in mean corpuscular volume in birds fed contaminated feeds [116].

ALT, AST, GGT, and other hepatic biomarkers increase following exposure to mycotoxins, especially aflatoxins, fumonisins, and Fusarium toxin mixtures, whereas total protein and albumin often decrease [117].

Importantly, intestinal barrier disruption is closely linked to gut dysbiosis, creating a bidirectional gut–immune axis in which altered microbial composition further amplifies systemic inflammation and pathogen susceptibility in poultry [110, 118].

### Gastrointestinal changes and microbiome alterations

Mold fungi produce mycotoxins, including DON, ZEN, AFB1, OTA, and T-2 toxin, that initially interact with the GIT and can disrupt barrier integrity and epithelial morphology. These gastrointestinal alterations represent a key mechanistic pathway underlying systemic health consequences in poultry. Effects include villus shortening, alterations in goblet cells, and changes in Paneth cells in both poultry and mammals [119-121].

This exposure also induces gut dysbiosis characterized by shifts in dominant taxa, reduced microbial diversity, and altered metabolite profiles in broilers and experimental models [107, 122, 123]. Under chronic low-dose exposure scenarios typical of commercial feeds, microbiome alterations may develop gradually and persist over production cycles, thereby predisposing birds to necrotic enteritis, reduced feed efficiency, and increased susceptibility to secondary infections despite the absence of overt clinical mycotoxicosis [121, 124].

Resident gut microbes can metabolize some mycotoxins, including DON, FB1, and ZEN, into metabolites that modulate toxicity and inflammatory responses in toxin- and microbe-specific ways [125-127].

Fumonisins and DON reduce populations of immunomodulatory bacteria such as Candidatus Savagella and some Lactobacillus spp. These toxins are associated with increased Clostridium perfringens in the ileum, thereby predisposing birds to necrotic enteritis. Chronic or subclinical exposure to Fusarium toxins and AFB1 consistently decreases villus height and alters crypt depth, thereby reducing absorptive surface area and nutrient uptake [81, 120, 128].

DON promotes intestinal colonization by enterobacteria such as Campylobacter jejuni and supports prolonged persistence or translocation of pathogens during co-exposure, thereby linking microbiome shifts to risks for both animal and public health [124]. This interaction suggests that mycotoxins may indirectly contribute to pathogen proliferation and altered host–microbiome dynamics, potentially increasing susceptibility to zoonotic infections and complicating disease control within poultry production systems [128].

Chronic DON exposure modifies the gut resistome, decreasing certain tetracycline resistance genes while increasing vancomycin-resistant subtypes in cecal metagenomes of laying hens, suggesting that mycotoxins may reshape functional microbial traits beyond taxonomic composition.

Mycotoxin exposure elevates pro-inflammatory cytokines and oxidative markers in intestinal tissue, thereby aggravating epithelial damage and altering local immune responses [21, 129].

### Effects on reproductive function and development

Fungal species such as Fusarium, Aspergillus, and Penicillium produce mycotoxins that contaminate cereals and feed, potentially impairing reproductive function and hepatic detoxification in animals [130, 131]. Emerging Fusarium toxins, including beauvericin, enniatins, and moniliformin, are increasingly detected in cereals and have been linked to reproductive effects in both in vitro and in vivo studies, highlighting potential risks to reproductive health [132].

Recent reviews (2024–2025) suggest that emerging Fusarium metabolites, particularly beauvericin and enniatins, may exert estrogenic, cytotoxic, and embryotoxic effects via disruption of steroidogenesis, mitochondrial function, and oxidative balance, thereby raising concerns for avian reproductive physiology under multi-mycotoxin exposure [14, 15].

Although direct data for avian species remain limited, exposure to various mycotoxins is known to adversely affect reproduction and embryonic development, leading to embryonic mortality and developmental abnormalities [133, 134]. Consequently, mycotoxins in animal feeds pose a probable threat to avian reproductive success and embryo viability, thereby affecting oocyte quality and early embryonic development, consistent with broader mycotoxin literature [131, 132, 134]. Given the frequent co-occurrence of multiple Fusarium and Aspergillus toxins in commercial feeds, cumulative and synergistic reproductive toxicity remains an underexplored but potentially significant risk in modern poultry production systems [132].

### Observed reproductive effects

Observed reproductive effects include reduced fertility, decreased egg production, and lowered hatchability, significantly affecting poultry productivity [135].

Exposure to mycotoxins during critical developmental windows can lead to congenital malformations and impaired embryonic growth [136].

T-2 toxin, notable for its high toxicity, affects both male and female reproductive systems by disrupting hormone synthesis and causing structural damage to reproductive organs. It also poses a significant embryotoxic risk by interfering with offspring development during pregnancy [137].

## RESIDUES OF MYCOTOXINS, THEIR TRANSFER, AND IMPACT ON FOOD SAFETY

### Transfer of mycotoxins to edible tissues

Evidence supports the transfer of several mycotoxins from contaminated feed into edible poultry tissues and eggs, including OTA, AFB1, citrinin, fumonisins, and DON. OTA and AFB1 have been consistently detected in muscle, liver, and egg matrixes, demonstrating documented feed-to-tissue carry-over and associated target organ pathology [44, 109, 122]. Chronic dietary exposure to citrinin results in accumulation in broiler muscle and layer tissues, including eggs, accompanied by histopathological alterations [95]. Fumonisins and DON have been associated with egg contamination and gastrointestinal lesions in young birds, highlighting the direct translation of feed contamination into edible products [41].

Collectively, these findings underscore the importance of feed monitoring and implementation of measures to reduce mycotoxin levels to protect poultry-derived food products. Moreover, the frequent co-occurrence of multiple mycotoxins in feeds increases the likelihood of cumulative carry-over into edible tissues, thereby reinforcing concerns regarding chronic low-dose dietary exposure and long-term food safety implications for consumers [138].

Studies demonstrate that broiler tissues can harbor multiple mycotoxins, with one investigation detecting 23 toxins in muscle, liver, and associated matrixes, thereby indicating systemic distribution [138]. OTA and AFB1 have been detected in eggs, including omega-3-enriched eggs, with OTA specifically identified in egg yolk [139, 140]. Aflatoxins have more broadly been reported in eggs across various studies, thereby highlighting persistent contamination risks [141]. Emerging mycotoxins in poultry feed and eggs further emphasize the ongoing risks to food safety [142]. In addition, masked and conjugated mycotoxins may persist in tissues and eggs while remaining undetected by conventional assays, potentially leading to underestimation of consumer exposure [57].

### Processing, cooking, and stability

An integrated strategy combining feed cleaning, processing, and post-harvest treatments can effectively reduce mycotoxin loads in poultry feed. Methods such as cleaning, milling, and extrusion can physically remove or degrade toxins, whereas post-harvest interventions help mitigate contamination at the source [131, 143, 144].

Thermal stability of major mycotoxins varies considerably. ZEN and OTA are relatively heat-stable. A concise summary of thermal degradation behavior of major and emerging mycotoxins during processing and cooking is presented in Table 2 [56, 57, 145–153] to facilitate rapid comparison of their stability and potential transformation products.

**Table 2:** Comparative thermal stability and transformation patterns of key mycotoxins.

Mycotoxin group	Typical thermal behavior in processing/cooking	Modified/masked forms and notes	References
Aflatoxins (AFB1, AFG1, etc.)	Partially destroyed at high temperatures; reduction increases with temperature, time, and moisture. Baking/roasting can produce approximately 40%–70% reduction in some systems, but complete destruction generally occurs only near approximately 250°C with prolonged exposure	Lactone ring opening and other degradation reactions; alkaline nixtamalization can reduce AFB1 by approximately 94% in maize, although some forms may reappear under acidic conditions	[57, 145-150]
OTA	Highly thermostable; minimal reduction under typical baking, frying, boiling, or short microwave conditions. Partial destruction occurs only near or above approximately 200°C or during intense microwave processing	Isomerization (e.g., 20R OTA) and other products may form during roasting/baking; toxicological profiles remain incompletely defined	[57, 145-150]
Fumonisins (FB1–3)	Thermolabile under intense heat, particularly with moisture or alkaline conditions; extrusion and nixtamalization can markedly reduce levels; complete degradation may occur above 180°C in some systems	Hydrolyzed and partially hydrolyzed fumonisins are often less toxic, although acidic conditions may reconvert some bound forms	[57, 146, 148, 149, 153]
DON and other trichothecenes	Moderately heat-stable; generally only modest reductions in baked products because crumb temperatures rarely exceed 100°C. High temperatures, prolonged baking/extrusion, or microwave treatment can reduce DON by >50% in some experimental systems	Conversion to less toxic isomers (e.g., isoDON) and deconjugation of deoxynivalenol-3-glucoside back to DON during steaming/fermentation	[145, 147, 150, 152]
ZEN	Relatively heat-resistant; baking and extrusion usually produce modest reductions; moisture exerts limited effects	Glucosides and sulfates occur naturally; many decrease by approximately 20%–50% during baking, with possible interconversion with free ZEN	[57, 147, 150, 152]
Alternaria toxins	Generally heat-stable; processing removes only limited amounts, emphasizing the importance of prevention strategies	Limited data are available regarding processing-induced forms; major research gap remains	[56]

Most major mycotoxins are chemically and thermally stable; therefore, common cooking practices only partially reduce them and frequently convert them into modified or masked forms rather than fully eliminating toxicity.

### Matrix and moisture effects

Degradation is usually greater in real food matrixes and at higher water activity. Water promotes hydrolysis of aflatoxins and some trichothecenes, particularly under microwave and extrusion conditions [145, 146, 147, 154].

Citrinin begins degrading around 100°C; however, in starch-rich foods it forms decarboxycitrinin and covalently bound carbohydrate adducts that are not detected in routine analyses but may be released during digestion [155, 156].

Similar carbohydrate or protein adducts have been demonstrated or inferred for T-2 toxin, fumonisins, and other toxins, thereby creating matrix-associated forms [57, 155, 156].

Plant-derived conjugates, including DON-3G, ZEN-14-Glc, and ZEN-14S, may both degrade into free toxins and be newly formed during fermentation and baking [57, 152].

### Non-thermal and innovative detoxification processes

Non-thermal and innovative processes such as ozone treatment, plasma technology, irradiation, and enzymatic detoxification can structurally degrade mycotoxins, often more efficiently than heating alone, although usually without complete decontamination and with uncertain toxicity profiles of resulting products [42, 145, 154, 157–160].

DON and patulin are more labile under high-temperature or alkaline conditions. Fumonisins and aflatoxins demonstrate intermediate losses depending on the matrix. Processing can reduce mycotoxin concentrations but often generates modified products whose toxicity and bioavailability require further investigation. Emerging and masked mycotoxins may follow distinct transformation pathways during processing, thereby generating metabolites with poorly characterized toxicity [142].

DON undergoes dehydration, lactonization, and cleavage, thereby forming norDON and related products, especially under alkaline conditions. Many of these compounds are reported to be less cytotoxic in vitro [161].

An additional research concern is the potential formation of transformation products and novel metabolites during biological, enzymatic, or chemical detoxification processes, whose toxicological profiles, bioavailability, and long-term safety in poultry and humans remain insufficiently characterized [38, 144].

Fumonisins can be hydrolyzed under alkaline conditions such as nixtamalization or react with reducing sugars and proteins during high-temperature processing, thereby forming adducts with uncertain toxicological profiles [146].

The initial mycotoxin concentration and the physical state of the feed, including whole grains, bran, or flour, influence degradation kinetics and observed degradation/half-life patterns during processing [162, 163].

Overall, conventional cooking and baking rarely eliminate mycotoxins completely; instead, they frequently redistribute, partially degrade, or convert them into modified or masked forms whose toxicity may sometimes be reduced, such as hydrolyzed fumonisins, isoDON, and decarboxycitrinin, but often remains insufficiently characterized. Prevention and targeted detoxification strategies therefore remain essential alongside processing interventions [153, 163].

Taken together, the thermal stability of mycotoxins varies considerably, and feed processing only partially deactivates these compounds, thereby emphasizing the importance of robust pre-harvest and storage prevention strategies to minimize contamination [149].

### Consumer exposure assessment and risk

Seasonal contamination of poultry feed can lead to fluctuations in mycotoxin concentrations in edible products, thereby indicating seasonal variations in consumer risk [46]. Residues of multiple mycotoxins have been detected in chicken breast and liver collected from market samples, confirming these concerns [164]. Direct dietary exposure to aflatoxins and OTA through chicken meat and eggs has been documented in urban areas of Cameroon [141]. These findings highlight that poultry-derived foods, particularly eggs and liver, may serve as important dietary exposure pathways in regions with high poultry consumption and limited feed quality-control systems [158].

Evidence of human exposure includes urinary biomonitoring studies demonstrating internal exposure to multiple mycotoxins in adults from high-consumption regions, including Europe and Africa, as well as population-level risk assessments highlighting substantial health risks associated with chronic multi-mycotoxin dietary intake [165, 166].

Nevertheless, occupational biomonitoring studies specifically targeting poultry farm workers, feed mill operators, and veterinarians remain extremely limited. Longitudinal biomonitoring using urinary and blood mycotoxin biomarkers could provide critical insights into chronic low-dose occupational exposure and cumulative internal burden under real farm conditions. However, long-term biomonitoring studies directly linking poultry-derived food consumption to internal mycotoxin biomarkers remain insufficient.

Integrated One Health-based exposure assessment frameworks combining dietary, occupational, and environmental data are therefore required to accurately characterize cumulative human exposure pathways within poultry production systems [19, 131, 167].

A comprehensive risk management system integrating feed safety, product testing, and mitigation measures can substantially reduce consumer exposure to mycotoxins and lower the burden of poultry product-related diseases [168]. Consumption of mycotoxin-contaminated poultry products may contribute to cumulative dietary exposure, thereby increasing long-term carcinogenic and genotoxic risks in exposed populations [169].

## OCCUPATIONAL AND ENVIRONMENTAL EXPOSURE IN POULTRY PRODUCTION

### Aerosolized spores and mycotoxins in air

Aerosolized fungal spores and mycotoxins in poultry houses pose substantial risks because dust originating from feed, litter, and manure can carry spores and toxins [30, 170]. Quantitative air sampling studies in intensive poultry facilities have detected measurable concentrations of airborne fungal spores, endotoxins, and mycotoxin-associated particulates in the inhalable dust fraction, indicating that occupational exposure may occur through chronic inhalation of contaminated aerosols during routine farm activities such as feeding, litter handling, and ventilation management [73, 74].

Studies show that spores and associated mycotoxins, including aflatoxins, OTA, and ZEN, are frequently present in poultry house air and originate from contaminated feed and litter [171-173]. Bioaerosol formation occurs at various stages of bird growth and is especially pronounced during winter ventilation [174].

Exposure to aerosols is associated with inflammatory responses in both humans and poultry, including upregulation of pro-inflammatory mediators [72, 73]. Despite increasing recognition of airborne fungal spores and mycotoxin-associated particulates in poultry houses, robust quantitative occupational exposure data remain scarce. Standardized exposure metrics, such as toxin mass per cubic meter of air, cumulative inhaled dose, and biomarker-based internal exposure assessment, are rarely incorporated into routine farm surveillance. Consequently, dose–response relationships, cumulative exposure duration, and internal biomarker profiles in poultry workers remain insufficiently characterized, thereby limiting comprehensive occupational risk assessment within a One Health framework [175-178].

An integrated One Health approach is therefore recommended to manage this risk because improved control of mycotoxin contamination at the farm and feed levels can simultaneously reduce animal health impacts, environmental fungal loads, and occupational respiratory exposure among farm workers [19, 165].

### Endotoxins, co-exposure, and complexity of bioaerosols

Poultry farms release complex bioaerosols in which dust is enriched with endotoxins and (1→3)-β-D-glucans, thereby stimulating pro-inflammatory responses in exposed workers [72, 73]. Comparative studies show that poultry houses accumulate the highest levels of total bacteria and fungi, whereas endotoxin and Gram-negative bacterial concentrations peak in pig farms, thereby illustrating diverse exposure profiles across livestock systems [74].

Dust from layer houses also contains high concentrations of endotoxins and extended-spectrum β-lactamase-producing enterobacteria, thereby posing risks to both birds and farm workers [179]. Health effects are confirmed by associations between bioaerosol exposure, lung function changes, and inflammatory markers during work shifts, with quantitative endotoxin assessment feasible during poultry handling tasks [180].

Because exposure is polymicrobial and multi-stressor in nature, the occupational exposome concept emphasizes co-exposures as key risk determinants and supports comprehensive monitoring and risk assessment in poultry production [181].

Within a One Health perspective, future research should prioritize structured occupational biomonitoring combining urinary and serum mycotoxin biomarkers, environmental air sampling, and seasonal exposure profiling to improve quantitative risk assessment at the animal–environment–human interface. Such cross-sectoral surveillance would enhance quantitative risk characterization and address current gaps in integrated occupational and environmental monitoring systems.

In this context, simultaneous exposure to bioaerosols, mycotoxins, endotoxins, and PM creates a complex inhalation risk profile that may exacerbate respiratory inflammation and long-term occupational health risks in poultry production environments. Adoption of an exposome-based One Health framework may therefore provide a more comprehensive understanding of occupational risks because poultry workers are simultaneously exposed to multi-mycotoxin bioaerosols, endotoxins, microbial pathogens, ammonia, and PM. Such cumulative multi-stressor exposure scenarios are rarely quantified and may exert synergistic effects on respiratory health, immune function, and long-term disease risk [14, 15, 131, 167, 181]. Mechanistic studies further indicate that co-exposure with oxidative agents and other pollutants can enhance airway inflammation, thereby highlighting the need for multicomponent risk models in poultry housing environments [182].

### Environmental spread and biosecurity

Residual litter, manure, and feed on poultry farms commonly serve as habitats for mycelial fungi, thereby creating environmental reservoirs that can negatively affect bird health. Mba and colleagues isolated fungal taxa from litter, feed, and fecal samples and demonstrated pathogenicity of selected isolates in day-old chicks, thereby highlighting the infection risks posed by these substrates [183].

Biosecurity in poultry production encompasses both external barriers that limit access by vehicles and unauthorized personnel, and internal procedures such as proper waste disposal, separation of clean and dirty zones, hygiene protocols, feed and water management, and routine cleaning and disinfection. These measures should be implemented in parallel with strict feed hygiene and storage management practices, as contaminated feed and litter are primary sources of fungal spores and airborne mycotoxins in poultry houses [184, 185].

Farm surveillance indicates that environmental samples, including litter, feed, water, and farm surfaces, can test positive for pathogens such as non-typhoidal Salmonella enterica, thereby underscoring the importance of biosecurity and waste handling [186]. Research on poultry pathogens such as Clostridium perfringens confirms the need for comprehensive biosecurity measures, adherence to hygiene standards, and prudent antimicrobial use to reduce infection risks and AMR associated with litter and manure reservoirs [187].

Effective biosecurity should also focus on air quality and incubator equipment, including monitoring for mycological contamination in feed and water, and surveillance of feed storage and water supply systems. Collectively, these findings underscore the necessity of integrated environmental, occupational, and feed management strategies within a coordinated One Health framework to minimize fungal proliferation, reduce occupational inhalation exposure, and limit downstream food safety risks in poultry production systems [103, 185].

## ANALYTICAL DETECTION AND MONITORING OF MYCOTOXINS

### Traditional methods

Traditionally, mycotoxin detection has long relied on chromatographic and immunoassay platforms [188, 189]. Chromatographic methods remain the backbone of confirmatory quantitative analysis. HPLC and LC-MS/MS are widely used for precise quantification. Further detailed discussion of LC-MS/MS and HRMS applications for multi-mycotoxin detection is provided in the subsequent subsection “Chromatographic and mass spectrometric methods” to avoid redundancy.

TLC and GC-MS are applied in specific analytical workflows. Automated LC-MS workflows, including robot-assisted sample preparation combined with LC-HILIC-MS/MS, are increasingly used, for example, in the detection of patulin in apple products [190, 191].

Immunoassays such as Enzyme-Linked Immunosorbent Assay (ELISA) and LFIA enable rapid screening. Widely used sample preparation strategies, including QuEChERS and immunoaffinity columns, are often combined with chromatographic methods to improve selectivity and sensitivity [188, 192].

Despite their central role, traditional methods may be costly, labor-intensive, environmentally sensitive, and require specialized technical expertise. These limitations have stimulated interest in ultrasensitive and portable alternatives such as ULISA and aptamer-based sensor platforms, which offer substantial potential for on-site rapid monitoring [192, 193].

### Immunoassays

Immunoassays remain key methods for mycotoxin detection because of their specificity, throughput, and cost-effectiveness across cereals, nuts, oils, and dairy products [190, 194, 195].

Recent advances have enhanced both sensitivity and multiplexing capabilities, including enzyme-loading cascade amplification for OTA detection [196], green aptamer-based ELISA [197], metal-organic framework cascade fluorescent ELISA for ZEN [198], and multiplex ELISA using 8–17 DNAzymes for simultaneous detection of multiple targets [199].

Ultrasensitive group-specific ELISAs now achieve pg/mL detection limits for AFB1 in diverse matrixes, including fermented feeds and feedstuffs, using matrix-optimized protocols [194, 200]. ELISA performance in complex matrixes such as table olives [201] and animal feeds [200], together with practical formats such as enhanced lateral flow assays [202] and capillary microfluidic ELISA for on-site testing [203], demonstrates strong field applicability.

Multiplex approaches, including ELISA with embedded calibration curves for aflatoxins, DON, and ZEN [204] and DNAzyme-based multiplex assays [199], further underscore the potential for high-throughput screening. Overall, ELISA continues to play a central role in mycotoxin analysis, supported by continuous advances in sensitivity, multiplexing, and practical field application.

### Chromatographic and mass spectrometric methods

Chromatographic and mass spectrometric platforms, particularly LC-MS/MS and UHPLC-MS/MS, dominate modern mycotoxin detection by enabling sensitive, selective, and multiplexed quantitative evaluation of food and feed matrixes [138, 205–207].

Although LC-MS/MS and UHPLC-MS/MS remain reference platforms for sensitive multi-analyte quantification of mycotoxins in food and feed matrixes, conventional targeted workflows may fail to detect masked, conjugated, and previously uncharacterized derivatives. Therefore, HRMS and non-targeted screening strategies are increasingly integrated to reveal the broader spectrum of hidden and emerging contaminants in complex poultry feed matrixes, particularly under multi-mycotoxin co-occurrence scenarios [14, 48, 206, 208, 209].

Sample preparation increasingly relies on QuEChERS-based extraction combined with mass spectrometric analysis, thereby covering broad toxin panels in feeds [210], maize/sorghum [211], and grains/spices [212]. Immunoaffinity cleanup with LC-MS/MS and isotope dilution further ensures reliability across matrixes, as validated in 11+Myco MS-PREP [213] and IAC–ID methods for six major toxins [214].

Real-world applications include cheese analysis for OTA and AFM1 [215], co-occurrence studies in rice bran and maize [216], and multi-mycotoxin detection in coffee [209]. These methods provide high sensitivity, selectivity, and multi-analyte capability, thereby making them indispensable for modern mycotoxin monitoring.

### Molecular and genomic tools

Molecular and genomic approaches are increasingly important for rapid and specific detection of mycotoxin-producing fungi and their toxigenic potential, thereby complementing traditional chemical analyses [217, 218].

CRISPR/Cas12a-based assays with isothermal amplification allow rapid field detection of Alternaria spp., a major mycotoxin producer, without requiring complex instrumentation [218]. Genome sequencing and analysis can reveal the presence or absence of mycotoxin biosynthetic gene clusters, thereby supporting risk assessment and differentiation of non-toxigenic strains [219].

Molecular detection of biosynthetic genes, combined with LC-MS-based biomarker approaches, facilitates both source attribution and human exposure assessment [208, 220]. Integration of omics-based tools with advanced mass spectrometry additionally supports identification of cryptic, masked, and previously uncharacterized mycotoxin metabolites that are increasingly recognized in complex feed matrixes [26, 221].

Nanotechnology-based platforms provide rapid and highly sensitive tools that complement PCR and sequencing for multiplex mycotoxin detection [217]. Overall, omics-integrated analytical frameworks and adaptive on-site detection strategies are increasingly recognized as key future directions in mycotoxin analytics.

### Biosensors and emerging technologies

Biosensors, particularly aptamer-based platforms, are increasingly used for the detection of mycotoxins such as AFB1, OTA, ZEN, and patulin because of their high affinity, stability, and cost-effective synthesis [222, 223].

A major advantage of biosensor platforms is their portability and suitability for on-farm and point-of-need testing, thereby enabling real-time monitoring of mycotoxin contamination in feed, litter, and environmental samples within poultry production systems [190, 222, 224].

Electrochemical transducers are widely used because of their portability, sensitivity, and rapid response time. Reviews highlight electrochemical, photoelectrochemical, and ECL methods [225-227]. Optical approaches such as fluorescent aptasensors and ECL provide exceptionally high sensitivity, including ECL assays for OTA and ZEN [224, 228].

Novel materials and analytical architectures, including MOFs [229], magnetic nanoparticles [230], carbon-based nanomaterials [231], paper-based origami sensors [232], and microfluidic devices [233], enhance portability and on-site testing capabilities.

CRISPR-Cas12a coupled with AIE amplification achieves ultrasensitive detection of gliotoxin and related mycotoxins [234].

Although traditional methods such as LC-MS/MS and ELISA remain reference standards for confirmatory analysis, biosensors and portable sensing platforms provide rapid on-site screening and real-time risk management tools that support integrated One Health surveillance across feed, environmental, and occupational exposure pathways in poultry production systems [131, 235, 236].

## MITIGATION AND CONTROL STRATEGIES

### Physical and chemical approaches

Physical and chemical detoxification methods are widely applied post-harvest to reduce mycotoxin contamination, although they may compromise nutrient quality and sensory properties [237, 238]. However, contemporary evidence suggests that single-intervention strategies are often insufficient under real-world conditions characterized by multi-mycotoxin contamination, masked forms, and climate-driven variability [160].

Physical approaches include sorting and washing, heating, irradiation, adsorption, non-thermal methods such as cold plasma, and storage control, including temperature, humidity, and gas composition [238-240].

Chemical strategies include alkaline treatment, ozone, chlorine dioxide, and ammoniation. Although these methods can degrade mycotoxins, they may also alter food/feed quality or leave residues [238, 241, 242]. Data indicate considerable variability depending on the matrix and toxin type. Moreover, conventional physical and chemical detoxification methods may show reduced efficacy against masked and emerging mycotoxins, which can remain structurally modified yet biologically active after processing and subsequently release parent toxins during digestion [4, 49]. For example, heating to approximately 250°C for 10 min can reduce AFB1 concentrations by up to 58%, whereas chemical methods may achieve substantial reductions under specific conditions [243].

Integrated strategies combining physical, chemical, and biological methods, often with the addition of probiotics or adsorbents, demonstrate synergistic detoxification potential [244]. Particularly promising are multi-hurdle strategies targeting complex toxin mixtures and masked mycotoxins by combining adsorbents, enzymatic detoxifiers, microbiome modulators, and phytogenic bioactives to achieve broader spectrum mitigation than single-component interventions. Such integrative approaches better reflect realistic contamination scenarios in modern poultry feeds [14, 15].

### Biological and enzymatic detoxification

Biological detoxification of mycotoxins in poultry feed relies on host metabolism, microbial biotransformation, and enzymatic degradation to reduce toxin bioavailability and tissue accumulation [45, 237, 245]. Emerging biotechnological approaches, including engineered microbial strains and CRISPR-assisted optimization of detoxifying enzymes, are being explored as next-generation tools for targeted degradation of complex mycotoxin mixtures. These precision biocatalytic systems may offer higher specificity and efficiency compared with conventional detoxifiers, particularly against emerging and modified toxins [154, 237, 238].

Bile acids enhance hepatic detoxification and excretion of AFB1 in broilers, representing a host-mediated mechanism [98]. Specific enzymes target ZEN, offering dedicated detoxification pathways [246]. Microbial degradation in the gut reduces OTA levels in tissues [237]. Microbial and enzymatic pathways convert DON into less toxic metabolites, including DOM-1 [247, 248].

Biological strategies also include probiotics and microbial binders. Combinations of Lactobacillus spp. and Saccharomyces cerevisiae with detoxifying agents improve performance and immune response in DON-exposed broilers [249]. Red yeasts, such as Sporidiobolus pararoseus, can act as mycotoxin binders, potentially reducing hepatic absorption in laying hens [250]. Multicomponent detoxifying agents combining various biological components are effective against AFB1 and T-2 toxin in broilers [251].

Nevertheless, the efficacy of current detoxifiers against emerging and modified mycotoxins remains insufficiently validated. Field performance is often assessed against single toxins, whereas their effectiveness under realistic multi-mycotoxin contamination scenarios, including emerging Fusarium metabolites such as enniatins and beauvericin, requires further large-scale validation. In addition, economic feasibility, scalability, and cost–benefit performance remain insufficiently evaluated, particularly in low- and middle-income poultry production systems [14, 249, 251].

Supplementary compounds further illustrate biologically mediated detoxification. Protocatechuic acid detoxifies FB1 in poultry [159]. Selenium, as selenomethionine, mitigates OTA-induced hepatic and renal transcriptomic changes [94]. Reviews also summarize enzymes that degrade ZEN and strategies for detoxifying OTA and DON, highlighting the biomolecular emphasis in poultry detoxification [127, 246–248].

### Phytogenic and feed-based strategies

Integrated phytogenic and feed-based strategies for mycotoxin detoxification in poultry feed combine plant-derived additives with adsorbents, functional additives such as enzymes, vitamins, and minerals, and microbial interventions to reduce toxin bioavailability and toxicity [102, 252].

Phytogenic additives provide antioxidant and anti-inflammatory protection and can modulate the gut microbiota, thereby increasing resilience to mycotoxins. Compounds such as luteolin and polyphenols are highlighted as detoxifying agents [250, 253].

Synergistic combinations of plant-derived bioactives with mineral or biological binders represent an innovative strategy to mitigate multi-toxin exposure, as phytochemicals may enhance gut barrier integrity and antioxidant defenses, while binders reduce systemic toxin absorption. This dual-mode approach is particularly relevant for chronic low-dose and co-contamination scenarios [14, 15, 84].

Adsorbents, including bentonite, clinoptilolite, nanosilica, and opoka, sequester mycotoxins [254, 255]. Products such as MMDA and Opoka have shown efficacy against OTA [252, 256, 257]. Synergistic combinations of probiotics and yeasts with detoxifying agents, phytogenics, and adsorbents enhance detoxification efficacy in contaminated diets [249]. In co-contamination scenarios involving AFB1, OTA, DON, and FB1, integrated phytogenic and adsorbent strategies mitigate adverse effects and enhance resilience [109].

In addition, modified veterinary mineral sorbents have been developed that can bind toxic metabolites and potentially reduce the risk of mycotoxicosis in poultry production systems.

### Preventive management and the One Health approach

An integrated One Health framework for poultry should combine feed hygiene, regular mycotoxin monitoring, and targeted nutritional interventions to protect growth, immunity, vaccine efficacy, animal welfare, and the safety of poultry-derived food products [10, 258].

Within the One Health context, emerging and masked mycotoxins can be conceptualized as hidden hazards that link environmental contamination in feed and crops, animal exposure through chronic ingestion, and human risk through residues in eggs, meat, and bioaerosols. This creates a transboundary exposure pathway often overlooked in traditional toxin-focused assessments [9, 216, 258].

Despite their clear relevance to food safety and environmental health, mycotoxins remain underrepresented within the broader One Health research landscape. Recent bibliometric analyses of more than 6,000 One Health publications indicate that topics such as zoonotic diseases and AMR dominate the field, whereas chemical hazards, including mycotoxins and pesticide residues, receive comparatively limited attention despite their alignment with UN Quadripartite Action Track 4 on food safety and environmental contamination [14, 15, 71, 131, 172].

Given the co-occurrence of mycotoxins and coccidiosis, holistic strategies, including strict feed control, disease prevention, and regular monitoring, are essential for maintaining health and productivity [21, 258]. Worker health must be prioritized because airborne mycotoxin exposure requires risk assessment and protective measures aligned with environmental, occupational, and food safety management protocols [167, 259].

Despite their relevance, mycotoxins remain an under-recognized occupational hazard within the broader One Health research agenda, where greater emphasis is often placed on infectious diseases and AMR, whereas chemical-biological co-exposures in livestock environments receive comparatively less quantitative investigation [16, 181, 185].

Future mitigation frameworks are expected to integrate artificial intelligence, IoT-based environmental monitoring, predictive analytics, and precision farming tools to enable proactive, real-time mycotoxin risk management across feed storage, transport, and poultry production systems. Such digitalized and data-driven approaches may enhance early detection, targeted intervention, and sustainable mitigation under climate-sensitive contamination scenarios, thereby aligning mycotoxin control with broader One Health and sustainability objectives [258, 260–263].

**Figure 2:**
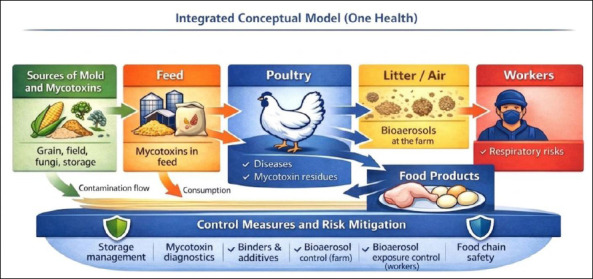
Integrated One Health conceptual model of mold and mycotoxin exposure pathways and risk mitigation in poultry production.

In accordance with the aforementioned considerations, an integrated One Health conceptual framework was developed to delineate the pathways of mold and mycotoxin exposure and identify key risk mitigation strategies within poultry production systems (Figure 2). This model illustrates the interconnected One Health dimensions of mold and mycotoxin contamination within poultry systems. Primary fungal contamination originates from crops in the field and during storage, leading to mycotoxin accumulation in feed. Following ingestion, poultry experience adverse health outcomes, including disease, immunosuppression, performance depression, and accumulation of mycotoxin residues in edible tissues and eggs. Concurrently, fungal spores, fragments, and toxin-bearing particulates from litter and the farm environment contribute to bioaerosol formation, creating occupational respiratory risks for workers. These combined processes extend beyond animal health and ultimately influence public health through potential carry-over into the human food chain. The lower panel highlights key intervention points and control strategies, including optimized storage management, routine mycotoxin diagnostics, application of binders and detoxifying additives, bioaerosol control at the farm level, mitigation of worker exposure, and comprehensive food chain safety measures. Together, these elements underscore the need for integrated surveillance, risk assessment, and coordinated multi-hurdle control strategies within a One Health framework, particularly to address multi-mycotoxin co-contamination, masked toxin forms, and occupational exposure risks in poultry production systems [131, 172, 181, 189, 258].

### Future research directions

Future research should prioritize large-scale, multi-farm validation of broad-spectrum detoxification strategies under realistic commercial poultry conditions, including feeds contaminated with multi-mycotoxin mixtures and masked forms. Particular attention should be given to the safety assessment of detoxification by-products and efficacy against emerging and modified mycotoxins.

Omics-based approaches, including metabolomics, mycotoxinomics, and microbiome profiling, are needed to identify unknown metabolites, secondary transformation products, and gut–liver axis responses to chronic exposure and mitigation interventions.

Integrated, multi-level strategies that combine feed hygiene, precision diagnostics, microbiome-targeted additives, and adaptive detoxification technologies will be essential for managing the evolving complexity of mycotoxin contamination under climate change and intensive poultry production conditions.

## RESEARCH GAPS

Despite significant progress in understanding fungal contamination and the impact of mycotoxins on poultry production, several critical knowledge gaps remain. Most existing data are derived from single toxin studies, whereas real poultry diets typically contain complex mixtures. Moreover, a major limitation of current toxicological research is the predominance of high-dose experimental models, which do not adequately reflect real-world poultry production conditions characterized by chronic, low-dose, multi-mycotoxin exposure below regulatory thresholds. Little is known about synergistic or additive effects, dose–response relationships under co-exposure, or cumulative risks under field conditions.

### Subclinical and real-world exposure scenarios

Increasing evidence indicates that poultry production systems are predominantly characterized by chronic exposure to low-to-moderate concentrations of multiple co-occurring mycotoxins, often below current regulatory or guidance limits. Unlike high-dose experimental models, these realistic exposure scenarios may lead to cumulative and subclinical effects, including impaired feed efficiency, gut dysbiosis, reduced vaccine responsiveness, immunomodulation, and increased susceptibility to enteric diseases such as necrotic enteritis. Recent meta-analyses and field-based studies presented in contemporary poultry science forums emphasize that current no-observed-adverse-effect level and lowest-observed-adverse-effect level thresholds derived from single toxin laboratory trials may not accurately reflect multi-toxin exposure under commercial conditions.

Current regulatory and guidance limits are largely based on single toxin assessments and vary substantially between regions. There is a need for globally harmonized, mixture-aware risk thresholds that reflect realistic multi-mycotoxin exposure scenarios and updated toxicological evidence from chronic low-dose studies. Notably, accumulating evidence suggests that prolonged exposure to mixtures of mycotoxins at concentrations considered individually safe may still result in additive or synergistic adverse effects, challenging current regulatory frameworks that are largely based on single toxin risk assessment. Long-term cohort studies on farms linking exposure to physiological and productivity parameters are largely absent.

### Masked, modified, and emerging mycotoxins

The identification of conjugated and modified toxin forms remains limited, and their toxicological significance, bioavailability, and impact on tissue residues are insufficiently studied. In particular, poultry-specific toxicokinetic data on masked and emerging mycotoxins, including absorption, hydrolysis, metabolic transformation, and residue transfer to eggs and meat, remain critically scarce, limiting accurate risk assessment under realistic feeding conditions.

Special emphasis is warranted for emerging Fusarium and Alternaria metabolites, including enniatins, beauvericin, moniliformin, and alternariol, whose increasing detection in poultry feeds contrasts with the scarcity of avian-specific toxicological and toxicokinetic data. Moreover, the toxicokinetics of masked and emerging mycotoxins in poultry remain poorly characterized, including their absorption, biotransformation, tissue distribution, and potential reconversion into parent toxins in the gastrointestinal tract, which may lead to underestimation of internal exposure and food safety risks.

### Occupational exposure and One Health surveillance gaps

Mycotoxins and fungal aerosols in poultry houses remain underinvestigated, and their effects on respiratory organs and overall worker health have not been quantitatively assessed. Future research should prioritize quantitative occupational exposure assessment for poultry workers, including standardized air sampling, personal exposure monitoring, biomarker-based monitoring, and dose–response modeling. Integrating these data into cross-sectoral One Health surveillance systems would significantly improve risk characterization at the animal–environment–human interface.

In addition, cross-sectoral surveillance systems integrating veterinary diagnostics, food safety monitoring, occupational health assessment, and environmental contamination data remain fragmented. The absence of harmonized One Health surveillance platforms for chemical hazards such as mycotoxins limits coordinated risk management and early warning capacity at regional and global levels.

### Food safety and tissue residue assessment

Comprehensive data on the transfer of mycotoxins and metabolites into eggs and meat remain inadequate for reliable food safety assessment. Future studies should evaluate the carry-over of regulated, masked, modified, and emerging mycotoxins into poultry-derived food products under realistic chronic exposure conditions. Such studies should also assess how feed composition, bird age, production type, toxin mixture, and mitigation strategy influence residue accumulation and consumer exposure risk.

### Mitigation validation and sustainability assessment

Although many binders, enzymes, and biological agents show efficacy in controlled experiments, real-world multi-farm evaluations are scarce, particularly those assessing economic aspects and integration across multiple exposure-reduction steps. Few studies integrate contamination scenarios into full cradle-to-farm-gate LCAs to quantify cumulative greenhouse gas emissions, nitrogen losses, water use, and land-use impacts associated with reduced performance and compensatory feeding. Future research should prioritize scenario-based LCA modeling to compare contaminated and mitigated feed systems and to inform evidence-based policy decisions on permissible limits and preventive investments.

### Analytical standardization and advanced monitoring

Variability in extraction protocols, detection methods, and reporting formats hinders global comparisons. There is a pressing need for internationally standardized multi-mycotoxin LC-MS/MS protocols capable of simultaneously detecting regulated, emerging, and masked forms with validated performance characteristics across diverse feed matrixes. Integration of non-targeted HRMS and omics-based workflows will be essential to detect masked and previously unrecognized mycotoxins.

### Longitudinal field studies and realistic safety thresholds

Particular priority should be given to longitudinal, field-based investigations conducted under commercial poultry production conditions, focusing on chronic low-dose exposure to multiple co-occurring mycotoxins. Establishing realistic no-observed-adverse-effect level and lowest-observed-adverse-effect level values for multi-toxin mixtures is essential because current regulatory benchmarks are largely derived from single toxin experimental models that may not reflect real-world feeding scenarios. Integrative studies assessing cumulative effects on nutrient digestibility, gut health, immune competence, vaccine efficacy, and long-term productivity are urgently needed to redefine safety thresholds for modern poultry feeds.

### Hidden hazard framework for poultry One Health systems

Conceptually, masked and emerging mycotoxins should be viewed as a unifying hidden hazard framework within poultry One Health systems, where undetected feed contamination leads to chronic subclinical exposure, potential residue transfer to animal-derived foods, occupational inhalation of contaminated dust, and cumulative public health risks. This perspective shifts the focus from regulated single toxin monitoring toward holistic multi-toxin and non-targeted surveillance strategies aligned with modern One Health risk assessment.

### Predictive modeling and climate-adaptive risk management

Future research should adopt a predictive modeling framework that integrates climate projections, meteorological data, trends in crop contamination, and poultry production parameters to forecast mycotoxin outbreaks under changing environmental conditions. Weather-based risk forecasting models, increasingly used in global feed monitoring programs, offer promising tools for early warning systems and proactive mitigation in poultry production. Such interdisciplinary approaches would enable early warning systems, proactive feed sourcing decisions, and climate-adaptive risk management before contamination reaches critical thresholds.

Addressing these knowledge gaps requires a transition from reactive detoxification toward predictive, integrated, and precision-based mitigation paradigms that combine climate-informed forecasting, advanced analytics, longitudinal field validation, and One Health-oriented surveillance to safeguard poultry health, food safety, and environmental sustainability.

## CONCLUSION

Mycotoxins remain a major and evolving threat to poultry production systems worldwide, affecting animal health, productivity, food safety, occupational exposure, and environmental sustainability within a unified One Health framework. The present review synthesizes recent evidence demonstrating that poultry feeds are consistently contaminated with multiple co-occurring mycotoxins, particularly aflatoxins, DON, fumonisins, OTA, and ZEN, while emerging and masked mycotoxins are increasingly detected in modern feed systems. Current evidence indicates that contamination is strongly influenced by climatic variability, fungal ecology, storage conditions, and global feed supply dynamics, leading to persistent exposure risks across poultry production environments.

The reviewed studies collectively demonstrate that chronic and multi-mycotoxin exposure contributes to hepatotoxicity, nephrotoxicity, oxidative stress, gut dysbiosis, immunosuppression, impaired vaccine responsiveness, reduced growth performance, compromised reproductive function, and tissue residue accumulation in poultry. In addition, aerosolized fungal spores and toxin-associated particulates within poultry houses represent an under-recognized occupational hazard for farm workers and veterinarians. Increasing evidence also suggests that masked and modified mycotoxins may evade conventional detection while remaining biologically active after gastrointestinal hydrolysis, thereby contributing to underestimated exposure and food safety risks.

A major practical implication of these findings is that conventional single toxin management approaches are insufficient under realistic commercial poultry conditions characterized by chronic low-dose exposure to complex mycotoxin mixtures. Therefore, integrated mitigation strategies combining feed hygiene, environmental monitoring, optimized storage management, adsorbents, biological detoxifiers, phytogenic additives, microbiome-targeted interventions, and precision analytical surveillance are essential for sustainable poultry production. The integration of LC-MS/MS, HRMS, biosensors, IoT-based environmental monitoring, and predictive analytics offers promising opportunities for early detection and proactive risk management in climate-sensitive production systems.

One of the major strengths of this review is its comprehensive integration of recent evidence from fungal ecology, toxicology, occupational exposure, food safety, environmental sustainability, analytical detection, and mitigation strategies, all within a One Health perspective. The review additionally highlights the growing significance of emerging, masked, and modified mycotoxins as hidden hazards in poultry systems and emphasizes the importance of multi-mycotoxin contamination scenarios rather than isolated toxin exposure. Furthermore, the inclusion of climate-related risk dynamics, occupational bioaerosol exposure, and sustainability implications broadens the current understanding of mycotoxin-associated risks beyond conventional feed toxicology.

Nevertheless, several limitations remain within the available literature. Most toxicological studies continue to rely on controlled high-dose experimental models rather than realistic chronic field exposure scenarios involving multiple toxins. Poultry-specific toxicokinetic data for emerging and masked mycotoxins remain limited, and standardized global surveillance systems integrating food safety, occupational health, and environmental monitoring are lacking. In addition, quantitative data regarding cumulative inhalation exposure, long-term biomonitoring, and the safety of detoxification by-products remain insufficient.

Future research should prioritize longitudinal field-based investigations under commercial poultry conditions, focusing on chronic low-dose multi-mycotoxin exposure, realistic mixture toxicity, and the establishment of updated mixture-aware safety thresholds. Greater emphasis should also be placed on omics-based profiling, non-targeted HRMS approaches, microbiome–gut–liver axis interactions, predictive climate-linked risk modeling, and integrated One Health surveillance systems. Validation of broad-spectrum detoxification strategies under multi-farm conditions, together with economic and sustainability assessments, will be critical to translating experimental mitigation approaches into practical applications in the poultry industry.

In conclusion, mycotoxin contamination in poultry production should no longer be viewed solely as a feed quality issue but rather as a complex One Health challenge involving interconnected animal, human, occupational, and environmental health dimensions. The increasing prevalence of multi-mycotoxin contamination, masked toxins, and climate-driven fungal shifts underscores the urgent need for integrated surveillance, precision diagnostics, predictive risk management, and coordinated multi-hurdle mitigation strategies. Strengthening these approaches will be essential to protect poultry productivity, food safety, worker health, and environmental sustainability under modern intensive poultry production systems.

## DATA AVAILABILITY

The data generated during the study are included in the manuscript.

## AUTHORS’ CONTRIBUTIONS

NM: Conceptualization, literature search, and writing – original draft, supervision. BN: Literature search and writing – original draft. AK: Literature analysis, visualization, and writing – review and editing. GN: Writing – review and editing, critical revision. ZK: Supervision and writing – review and editing. All authors have read and approved the final version of the manuscript.
